# Factors associated with hospital readmission in sickle cell disease

**DOI:** 10.1186/1471-2326-9-2

**Published:** 2009-02-27

**Authors:** Monique Morgado Loureiro, Suely Rozenfeld, Marilia Sá Carvalho, Rodrigo Doyle Portugal

**Affiliations:** 1 Rio de Janeiro Federal University (UFRJ), Rio de Janeiro, RJ, Brazil; 2 National School of Public Health (ENSP), Oswaldo Cruz Foundation (Fiocruz), Rio de Janeiro, RJ, Brazil

## Abstract

**Background:**

Sickle cell disease is the most frequent hereditary disease in Brazil, and people with the disease may be hospitalised several times in the course of their lives. The purpose of this study was to estimate the hazard ratios of factors associated with the time between hospital admissions.

**Methods:**

The study sample comprised all patients admitted, from 2000 to 2004, to a university hospital in Rio de Janeiro State, south-east Brazil, as a result of acute complications from sickle cell disease (SCD). Considering the statistical problem of studying individuals with multiple events over time, the following extensions of Cox's proportional hazard ratio model were compared: the independent increment marginal model (Andersen-Gill) and the random effects model.

**Results:**

The study considered 71 patients, who were admitted 223 times for acute events related to SCD. The hazard ratios for hospital readmission were statistically significant for the prior occurrence of vaso-occlusive crisis and development of renal failure. However, analysis of residuals of the marginal model revealed evidence of non-proportionality for some covariates.

**Conclusion:**

the results from applying the two models were generally similar, indicating that the findings are not highly sensitive to different approaches. The better fit by the frailty model suggests that there are unmeasured individual factors with impact on hospital readmission.

## Background

In Brazil, sickle cell disease (SCD) is the most prevalent of the hereditary diseases, particularly among Afro-descendants. It is characterised by mutation of the beta globin chain gene, producing an abnormal variant called haemoglobin S (HbS) [1]. Abnormal haemoglobin can cause various clinical events, the most common being vaso-occlusive crisis (VOC), characterised by acute episodes of abdominal, thoracic and bone pain and chronic haemolytic anaemia. These events, particularly VOC, require hospital care [2,3].

The disease evolves very differently from patient to patient and this variability seems to be related to hereditary and environmental factors, including aspects of socio-economic situation. While the disease progresses benignly and even without symptoms in some patients, others are more prone to complications, and a small subgroup has high rates of VOC and requires multiple hospital admissions [4]. Several studies estimate that high admission rates are associated with worse prognosis and higher mortality [5-7].

Sickle cell disease causes premature death [8], particularly in Brazil where, from 1996 to 2000, more than 60% of deaths from the disease occurred in patients under 29 years old [9]. A North American study estimated that the probability of death among 10 to 19 year olds with SCD was 8 times higher than for the population of the same age and race [5]. High number of symptoms is associated with worse prognosis and early death. Patients with SCD who undergo a large number of painful episodes are more likely to die prematurely [8].

Hospital care generally has been an important object of health service research, given its central role in care and high costs. Knowledge of factors associated with hospital service use is crucial to public policy-making to improve these services in Brazil [10]. This approach should be applied with the focus on specific diseases, especially those in which patients need multiple hospital admissions, as is the case with SCD. The purpose of this study is to estimate the effect of factors associated with successive hospitalisations of patients with SCD.

## Methods

This is a retrospective study of SCD patient records. The sample studied included all the patients admitted to a public university hospital over a five-year period (2000–2004) as a result of acute events related to SCD. The hospital, located in Rio de Janeiro City in south-east Brazil, forms part of the national health system (*Sistema Único de Saúde*) and is a reference centre for the treatment of adolescents and adults with SCD. Patient records were identified by the diagnoses that appear in the hospital's electronic records. The patient records selected were those bearing D57 codes (sickle cell disease), according to the International Classification of Diseases – Tenth Revision. After examining the records, those of patients not admitted during the study period or with sickle-cell trait were excluded. All non-elective admissions for acute events, in which patients remained in the hospital for at least 24 hours, were included. Patients who died during the first hospitalisation were excluded. A form and an instruction manual were developed to guide extraction of information on the patients and their admissions from the patient records. All readmissions of each patient were considered for analysis, regardless of the time elapsed between them.

Time to readmission or to censoring by death or end of the observation period was modeled considering demographic and clinical covariates. Demographic covariates included were age, gender, ethnicity and education. The following independent clinical covariates were evaluated: age, phenotype, vaso-occlusive crisis, acute thoracic syndrome, bacterial infection, chronic renal failure (CRF), length of hospital stay (in days), use of opiates and number of packed red blood cell transfusions. The clinical covariates were selected on the basis of clinical criteria considered relevant in SCD patient admissions.

The statistical approach was based on extensions of Cox's proportional hazard model. Two models were fitted, one marginal and the other conditional or of random effects [11]. The Cox survival model is defined as a semi-parametric model because the baseline hazard function is treated non-parametrically, not assuming any specific probability distribution, while a parametric form is assumed for the covariate effects. It is also called a proportional hazards model as the ratio of hazard rates of any two individuals is proportional and constant over time. However, when an individual can experience multiple events, the dependency between events within individual has to be taken into account, and extensions of this model have been proposed.

Among the various options, the Andersen-Gill (AG) model, also known as the independent increment model was chosen. Independence here means that each event – "event" being each patient's hospital admissions – is not conditioned by previous ones, i.e. by previous admissions. That is, after each admission has occurred, the risk of admission returns to the prior situation, depending only on the characteristics of the individuals involved. The events share the same covariates, but are independent of one another.

Mathematically, the definition of the AG model is similar to the classic Cox proportional hazard model:

$$\lambda_{it}(x_{i})=H_{it}\lambda_{0}(t)\exp(x_{i}^{,}\beta )$$

where *H*_*it *_indicates whether the subject *i *is at the time *t *at observation and/or risk (= 1) or not (= 0), *λ*_*o*_(*t*) is an arbitrary baseline hazard rate which remains unspecified, **β **is the vector of parameters associate to the **x **vector of covariates. The main difference between this model and the classical Cox model is the construction of the risk set – individuals at risk in each observed time – and the estimation of robust standard errors in the presence of repeated events. In the AG model, as soon as the observed event ends, the individuals is back to the same "at risk" situation.

Another approach to deal with multiple events is via random effects models. Suppose a random variable *z*_*i *_representing an unknown random effect, related to each patient, with unit mean and variance *ε*_*i*_. Large values of *ε*_*i *_reflect a greater degree of heterogeneity among patients. The form often assumed for the hazard rate is

$$\lambda_{it}(z_{i}x_{i})=(z_{i})H_{it}\lambda_{0}(t)\exp(x_{i}^{,}\beta )$$

The model holds two standard assumptions: (i) observations within each patient share a common frailty effect over time, (ii) conditional on an chosen parametric distribution – in this case a gamma distribution – the times to events are assumed to be independent.

The intra-individual correlation is thus corrected, and by means of this random effect, a set of unmeasured individual characteristics, which could be described as an individual "frailty", is estimated. Here, differently from the marginal models, the point estimate can be substantially altered, depending on the magnitude of this random effect.

Schoenfeld residuals were used to evaluate the assumptions of proportionality [12]. The explanatory power of the models was estimated using an R^2 ^indicator, calculated as 1 minus double the likelihood ratio between zero and the proposed model [11]. All the models were fitted using R software and the "survival" library [13].

The study was approved by the Research Ethics Committee of the National School of Public Health, Oswaldo Cruz Foundation (Rio de Janeiro).

## Results

From 2000 to 2004, the hospital treated 108 patients with sickle cell disease, of whom 78 were hospitalised at least once. Seven patients died during their first hospitalisation and for that reason were excluded. The remaining 71 patients underwent 223 hospitalisations, of which 88.0% (196) were readmissions, for treatment of SCD-related acute events. The sample mean age was 20.2 years (13–53). Most patients (85.9%) were admitted up to five times during the study period, 5 were admitted 10 or more times, and 1 patient was admitted 20 times. The median of admissions was 2.0 (1–20) and the most frequent cause was vaso-occlusive crisis (73.1%). The patients' characteristics are shown in Table 1.

**Table 1:** Distribution of patients characteristics and hospital admissions in patients with SCD admitted to a public university hospital, southeastern Brazil, 2000–2004

Patients Characteristics	Frequency (%)
N = 71	
	
Sex	
Male	36 (50,7)
Female	35 (49,3)
	
Phenotype	
SS	57 (80,3)
Others	14 (19,7)
	
Ethnicity	
White	14 (19,7)
Non-white	57 (80,3)
	
Education	
Elementary school	41 (57,7)
Other	30 (42,3)
	
Hospital admissions	
N = 223	
	
Vaso-occlusive crisis	163 (73,1)
	
Acute chest syndrome	17 (7,6)
	
Bacterial infection	64 (28,7)
	
Chronic renal failure	11 (4,9)
	
Opiate use	188 (84,3)

Table 2 shows the hazard ratios for hospital readmission, by characteristics of the patients and admissions, in two survival models. On both the AG and frailty models, the occurrence of vaso-occlusive crisis (VOC) in a prior admission associated significantly with time elapsed to the following admission. Likewise, in both models, chronic renal failure (CRF) associated significantly with time between admissions, although the greater risk was encountered on the frailty model (RR = 27.25 CI 5.11- 145.43). None of the demographic characteristics were associated with time to readmission either on the AG or on the frailty models.

**Table 2:** Comparison between survival models in hospital readmissions in patients with SCD admitted to a public university hospital, southeastern Brazil, 2000–2004

Clinical characteristics				
	*AG model*		*Frailty Model*	

	HR	95% CI	HR	95% CI
Age	0.99	0.96–1.02	0.98	0.94–1.01
				
Phenotype				
Others	1.00		1.00	
SS	1.60	0.92–2.77	1.96	0.88–4.37
				
Vaso-occlusive crisis				
No	1.00		1.00	
Yes	2.06	1.23–3.45	2.14	1.19–3.87
				
Acute chest syndrome				
No	1.00		1.00	
Yes	1.30	0.54–3.11	1.23	0.56–2.73
				
Bacterial infection				
No	1.00		1.00	
Yes	0.90	0.54–1.51	0.89	0.51–1.53
				
Chronic renal failure				
No	1.00		1.00	
Yes	22.17	10.13–48.54	27.25	5.11–145.43
				
Opiates				
No	1.00		1.00	
Yes	1.32	0.62–2.84	0.70	0.33–1.46
				
Number of red cell concentrate transfusions	1.04	0.86–1.26	1.03	0.87–1.23
				
Length of hospital stay (days)	0.99	0.96–1.02	1.00	0.96–1.03
				
Frailty Variance			0.85	p = 0.00000

AG = Andersen-Gill; HR = hazard ratio

The models' explanatory power evaluated by the R^2 ^measure, was 0.25 for the AG model and 0.60 for the frailty model, indicating a better fit by the latter, where the variance of the random effects was 0.85. In the frailty model, 3 patients were outliers, with excessively high hazard for hospital readmissions (data not shown).

The analysis of Schoenfeld residuals indicated serious limitations of the AG model. Covariates such as age, number of packed red blood cell transfusions and length of hospital stay on previous admission, although not significantly associated with risk of a further admission, presented linear correlation with time, suggesting that this model does not comply with the proportion hazard assumption.

## Discussion

It is important to identify what type of variable (demographic, genetic or clinical) and what variables are associated with the risk of hospital admission in patients with SCD, because some of them will undergo multiple hospitalisations in the course of their lives. The assumption of independence of hospital admissions means that after being discharged from hospital the patient returns to the baseline risk of a further hospitalization occurring. The reasoning that sustains this hypothesis is the acute characteristic of the events, that do not result in sequelae (at least, not "measurable" ones), and therefore are resolved during the hospital stay. In such a setting, the AG independent increment model is reasonably adequate.

Although the occurrence of successive VOCs causes chronic organ damage, such damage is slow to appear. That is why it is plausible to think, in studies with relatively short observation periods, that VOC behaves as an independent event at each readmission. As regards CRF, on the AG model, the estimates point to a significant association with time elapsed until readmission, and the hazard ratio is high. CRF is a difficult condition to compensate for, and is often associated with non-SCD-related complications that require hospital treatment. In addition, CRF associates with premature death in patients with SCD.

A number of prognostic factors for the occurrence of unfavourable outcomes and serious conditions from SCD have been described [14]. Foetal haemoglobin, the coexistence of thalassaemia and haplotypes have been studied much more as risk factors modulating these clinical outcomes, as have various genetic polymorphisms described recently [15]. The unfavourable outcomes generally require hospital treatment and it is possible that they influence the number of admissions and the time intervals between them, given that the frequency of hospitalisation also associates with worse prognosis and increased risk of death [6,7,16]. Presentation in multiple phenotypes – i.e. the great variability within SCD, a monogenic disease – is probably due to genetic factors that have not yet been completely studied.

In this context, the frailty model offers the advantage of improving the estimation of parameters and related standard errors, even in the absence of important covariates, as it contemplates the role of unmeasured variables, such as genetic factors. In the frailty model, the VOC and CRF variables remain significant, despite the large confidence interval. The hazard ratio for CRF was also very high, as in the AG model. The highest R^2 ^was observed in the frailty model (0.60), corroborating the importance of unmeasured factors. As yet undetermined genetic aspects, such as polymorphisms, may account for this great variability.

Other approaches were also considered, such as the Prentice, Williams and Peterson (PWP) model, in which each event belongs to a different, ordered stratum (first hospitalization, second hospitalization, and so on). Using time-dependent strata means that, unlike the AG model (where the likelihood of any event is the same), the underlying intensity function can vary from event to event. SCD is hereditary and its clinical manifestations occur from birth. Therefore, in order to fit a PWP model, it would be necessary to number and order each hospitalisation, which would require hospital records for a patient's whole life, which is beyond the scope of this study.

In the frailty model three patients displayed excess hazard significantly greater than 1, suggesting that in these cases the individuals had other characteristics which would explain the times to hospital readmissions. The three patients were young females. The patient with greatest relative hazard in the frailty model was the one most hospitalised in the period and, among all the patients in the hospital, the only one diagnosed as dependent on morphine, an opiate used to control pain in patients with SCD. Morphine dependence, although rare in the study sample, was certainly decisive in the pattern of hospitalisation, because it is a condition that causes greater need for the drug, leading the patient to seek hospital care more often. It is important to look for signs of opiate dependence, which should be treated promptly by appropriately trained personnel.

In this study, the models did not include others patients' socio-economic variables which might be associated with hospital re-admission, for lack of such information in the patient records. This is a limitation on the study, because it is reasonable to suppose that the patients' social profile would influence the pattern of hospitalisation. For example, some patients may face financial difficulties in purchasing drugs and thus seek hospital care more often, even when the pain could be controlled at home.

## Conclusion

In this study, we observed that the semi-parametric modelling of proportional hazard ratio was appropriate to studying multiple hospitalisations in patients with SCD. The fit of the AG model displayed limitations. The high R2 encountered in the frailty model suggests that factors not included in the modelling were important in the hazard of hospital readmission.

## Competing interests

The authors declare that they have no competing interests.

## Authors' contributions

MML contributed conception and design, data collection, data analysis and interpretation and preparation of the manuscript. SR contributed conception and design, data analysis and interpretation and review of the manuscript and approved the final version for publication. MSC contributed data analysis and interpretation and approved the final version for publication. RDP review of the manuscript and approved the final version for publication. All authors read and approvede the final manuscript.

## Pre-publication history

The pre-publication history for this paper can be accessed here:


